# ASEAN integration and health services

**DOI:** 10.3402/gha.v8.27199

**Published:** 2015-03-25

**Authors:** Jiruth Sriratanaban

**Affiliations:** 1 Thailand Research Center for Health Services System, Bangkok, Thailand; 2 Faculty of Medicine, Chulalongkorn University, Bangkok, Thailand

It seems to be an exciting moment for the ASEAN countries to enter the era of the ASEAN Community. For the ASEAN 600 million people (8.6% of the World population), this regional initiative is expected to bring about economic prosperity and development in socio-cultural well-being of the people. However, development policies do not always complement one another as we promote economic growth and improve quality of life of the people. As part of the ASEAN Social and Cultural Community (ASCC) to promote equity in healthcare access across the region, the progress towards universal health coverage (UHC) becomes a key challenge. Different countries are at different stages of UHC development (1). I agree with Hoang et al. highlighting three major constraints – including financial constraints, supply-side constraints and ongoing epidemiological transition. Nevertheless, the supply-side constraints, such as inadequate numbers of health workers, become my particular concern since the ASEAN Economic Community (AEC) movement includes promotion of different forms of trade in health services, ranging from exports of cross-border health care to migration of health professionals and direct foreign investment in the health sector. The liberalization of the health market raises demands for for-profit health services, competing for the same pool of resources needed for achieving UHC for the population of many ASEAN countries. Thailand, for example, has been facing a dilemma of promoting health services as an economic product under the medical-hub policy versus protecting health security of the people under the universal coverage policy. The growth of the private health sector has intensified competition in the labor market. The expansion of private health care has led to the rise in professional fees and salaries widening income gaps and causing the ‘Brain-drain’ problem in the public sector (2). Several public hospitals have faced high turnover of health personnel, particularly physicians and nurses (3).

The health coverage for migrants should also be focused. As the AEC would promote economic prosperity of the region, it could be expected that labor migration would continue. Cross-border health services and migrant workers – both documented and undocumented – could considerably increase service workloads for providers in the receiving countries. Although they might become income opportunities for some hospitals in certain well-to-do areas, the uninsured frequently result in uncompensated care in public hospitals (4, 5). The situation also compromises access to care and, thus, potentially affects health of this segment of the ASEAN population. Despite some countries’ efforts to address the issue of migrants’ health coverage (6), I foresee a long way to go – particularly for the undocumented migrants. The key question is ‘Who is, or are, going to pay?’ Some alternatives are compared and discussed in Table 1.

**Table 1:** Paying for health coverage of undocumented migrants

Potential payers	Rationale	Possible arguments
Undocumented (illegal) migrants themselves	Most justified as they earn income from work.	Don't forget that they are undocumented. Any legal insurance programs allow for their participation. Any incentives for them as they are in usually healthy working ages.
Documented migrants paying insurance premium	Special health coverage programs for migrants by receiving countries, or by sending countries.	Why the documented (legal) migrants have to pay for. Any incentives as they are in usually healthy working ages.
Employers of the undocumented migrants	Also justified as employers benefit from the hiring.	Don't forget about the undocumented status of the migrants. Why would any employers declare their hiring and pay? Any legal insurance programs allow for their participation.
Government of the receiving country	Benefit to the economy of the country from extra (and maybe cheap) labor force.	Is it legal? Can it be sustained? Is it politically acceptable? It has to be funded by ‘Tax money’.
Government of the sending country	To protect the citizen working aboard; some programs for documented migrants exist in a few ASEAN countries.	Is it politically an issue? Universal health coverage may not even exist for those residing in that country yet. Is it financially feasible for the undocumented group?

The ASEAN integration and its impacts on health services, particularly on health coverage for the population, is obviously a challenging situation. Given the diversity among the ASEAN countries, policies on health services and health coverage after the ASEAN integration should be carefully planned such that every nation can benefit. There are choices for approaching UHC in terms of whom to be covered, what services to be covered and at what level of payment. In light of the constraints, we may need building blocks and roadmaps that could align the region and each ASEAN country. Still, the key is collective commitment among the ASEAN members in income and wealth distribution, and health equity. To make it financially feasible for ASEAN, I would like to propose that ASEAN starts UHC with the coverage for selected basic health services for everyone, such as traffic accident and some epidemic-prone communicable diseases, rather than beginning UHC talk focusing on coverage for everything for selected groups, or UHC for all of the population. The step-by-step approach helps ensure the minimal standardized package of UHC for all ASEAN countries to address critical public health issues, allows for insurance portability, and makes it practical to manage reimbursement. This also helps address the issue of health coverage for undocumented migrants.
